# Oligozoospermic infertile sperm with high ROS are more prone to epigenetic alterations

**DOI:** 10.3389/frph.2026.1933266

**Published:** 2026-09-23

**Authors:** Anamika Kumari, Syed Waseem Andrabi, Rajender Singh

**Affiliations:** 1 Central Drug Research Institute, Lucknow, UP, India; 2 Academy of Scientific and Innovative Research (AcSIR), Ghaziabad, Uttar Pradesh, India; 3 Milan, The Fertility Clinic, New Delhi, India

**Keywords:** DNA methylation, epigenetic, male infertility, methylation, oligozoospermia, sperm

## Abstract

**Introduction:**

Oxidative stress has been found to play a critical role in sperm quality and function. In this study, we asked if oligozoospermic samples with high ROS display a higher degree of methylation alterations in comparison to normozoospermic samples.

**Methods:**

We investigated genome-wide DNA methylation in normozoospermic (presumptive fertile) and oligozoospermic infertile samples with high and low ROS levels. Fifty samples each for oligozoospermia and normozoospermia were analyzed for their ROS levels. Two samples each in the normozoospermic low ROS, normozoospermic high ROS, oligozoospermic low ROS and oligozoospermic high ROS categories were selected for genome-wide DNA methylation profiling.

**Results and discussion:**

A comparison between oligozoospermic and normozoospermic samples showed a low number of differentially methylated genes (about 100). The normozoospermic high ROS group in comparison to the normozoospermic low ROS group showed a moderate number of differentially methylated genes (538). On the other hand, oligozoospermic high ROS versus oligozoospermic low ROS comparison showed the highest number of genes (1,755) to be differentially methylated, of which a majority displayed hypomethylation. These findings underscore the vulnerability of oligozoospermic sperm to epigenetic disruptions induced by oxidative stress. We conclude that oligozoospermic samples with high ROS carry a high degree of methylation alterations.

## 1 Introduction

Infertility affects around 15% of couples globally and the incidence is rising rapidly (1). Out of the total infertility cases, about 40% are due to male factors, 40% are due to female factors, and 20% are due to mixed factors. The most common reasons for male factor infertility include stress (including oxidative stress), genital tract infections, endocrinological disorders, varicocele, ejaculatory failures, chromosomal abnormalities, and immunologic problems (2). Increased ROS, also labelled as oxidative stress, was recognized as a significant contributor to infertility long ago (3). Over the last four decades, research on human and animal models has firmly established oxidative stress as the major contributor to male infertility (3–5). Of all infertility cases, 80% are due to elevated ROS (1). ROS are required for various physiological processes, such as motility, hyperactivation, capacitation, and acrosome reaction, but their uncontrolled production is an important factor in various disorders, including infertility. High ROS levels have been suggested to impair fertility by favoring lipid peroxidation and DNA damage due to limited ROS scavenging capacity in spermatozoa (6).

In our previous studies, we showed that spermatozoa from infertile men have significantly altered DNA methylation (7, 8). Though the cause of altered DNA methylation in these samples remained unknown, all possible infertility-causing factors could underlie epigenetic modifications. The top contributor to infertility, i.e., high ROS, is well known to affect the level of DNA methylation enzymes (DNMT1, DNMT3A and MBD4) and ten-eleven translocation enzymes (TET1, TET2, TET3) (9, 10). In addition to the above, high ROS decrease glutathione (GSH) levels, which ultimately decreases S-adenosylmethionine (SAM), thus compromising DNA methylation. Apart from decreasing DNA methylation by the above mechanisms, high ROS also lead to the formation of 8-hydroxydeoxyguanosine (8-OHdG), which triggers conformational changes in chromatin, blocking the interaction of DNMTs with DNA. 8-OHdG also recruits OGG1 and TET1, initiating the DNA de-methylation pathway (9). In this way, high ROS can lead to significant demethylation, leading to changes in gene expression, affecting sperm production and fertility.

In the present study, we hypothesized that high ROS play a significant role in altering DNA methylation in male germ cells. To test this hypothesis, we compared sperm DNA methylation levels between oligozoospermic infertile men with low and high ROS and separately between normozoospermic men with low and high ROS levels. This helped us interrogate the impact of ROS on sperm DNA methylation in normozoospermic and oligozoospermic men.

## 2 Methods

### 2.1 Participants and sample collection

Semen samples were collected by masturbation as per the WHO recommendation after following the required abstinence period (2–4 days). The inclusion criteria for patients consisted of 25–45 years age group having primary infertility for more than one year and the exclusion criteria consisted of individuals with chromosomal abnormalities, hypogonadism, varicocele, infection of the genital tract, physical deformities, and any disease affecting reproductive functions. The normozoospermic group (presumptive fertile) consisted of men in the same age bracket with normal semen parameters as per the WHO 2021 criteria. The purpose of the study was disclosed to all individuals and informed written consent was obtained before participation. After liquefaction at 37 °C for 30 min, sperm parameters were assessed using Mekler chamber under a microscope. Semen analysis was undertaken on the basis of the WHO 2021 guidelines.

### 2.2 ROS estimation in sperm

We measured the intracellular reactive oxygen species (ROS) in sperm using the fluorescent probe H_2_DCF-DA (ab113851). After liquefaction, semen samples were washed using PBS at 200 g for 10 min, followed by somatic cell lysis buffer treatment as detailed previously (11). Samples were again centrifuged to obtain sperm pellet, followed by a PBS wash. Each sample was divided into three aliquots. The first aliquot was not incubated with fluorescent dye and served as a negative control. The second part was first incubated with 100 µM of H_2_O_2_ for 10 min, followed by incubation with the dye and served as a positive control. The third part of the sample was incubated with 10 µM of H_2_DCFDA at 37 °C for 30 min. After incubation, the samples were centrifuged to obtain sperm pellet. Sperm were resuspended in 500 μL PBS and were immediately assessed through flow cytometry (FACS-Aria) using H_2_DCFDA dye with excitation and emission at 485 nm and 535 nm with a flow rate of 300–400 cells/sec. The conversion of DCFH-DA into DCF was used to quantify ROS in sperm cells.

Based on the mean ROS value, both normozoospermic and oligozoospermic samples were divided into two categories, i.e., low ROS and high ROS. Samples with mean ROS value ≤500 were categorised as low ROS, i.e., normozoospermic low ROS (NLR) and oligozoospermic low ROS (OLR), while samples with mean ROS value ≥1,000 were categorised as high ROS, i.e., normozoospermic high ROS (NHR) and oligozoospermic high ROS (OHR).

### 2.3 DNA extraction

Semen samples were processed using differential centrifugation to obtain pure spermatozoa. First, the samples were allowed to liquefy, followed by a PBS wash. Afterwards, the samples were incubated with somatic cell lysis buffer for 30 min on ice to get rid of contaminating leukocytes. After the PBS wash, the supernatant was discarded and sperm were resuspended in 150 µL of PBS. The samples were further processed as per the protocol for DNA isolation using the MasterPure Complete DNA and RNA Purification Kit (Cat. No. MC85200).

To 150 µL of sample, 150 µL of 2X-T&C lysis buffer was added supplemented with 1 µL of proteinase K. The samples were incubated at 65 °C for 15 min with intermittent mixing. Following this, the samples were brought to 37 °C and 5 µg of RNaseA was added. After incubation at 37 °C for 30 min, the samples were shifted to ice for 5 min, following which 150 µL of the MPC reagent was added. This was followed by centrifugation at 14,000 g for 10 min, collection and transfer of the supernatant to a new vial. Chilled isopropanol (500 µL) was added to the supernatant, followed by incubation at room temperature for 10 min. The samples were centrifuged at 14,000 g for 10 min to obtain the DNA pellet. The pellet was washed with 70% ethanol twice by centrifugation. DNA was resuspended in 20 µL of nuclease-free water. DNA was visualized on 1% agarose gel, followed by quantification through the Qubit dsDNA high sensitivity fluorometric assay (Invitrogen, USA).

### 2.4 DNA library preparation and sequencing

The DNA library was prepared using the NEBNext Enzymatic methyl-seq kit (Cat. No. E7120). 50–100 ng spiked-in PUC19 and lambda DNA (provided in the kit) were fragmented to 250 bp using Covaris M220 (Covaris, USA). The fragmented DNA was then ligated with EM-seq adaptor, followed by oxidation of 5-methylcytosine to 5-hydroxymethycytosine using the TET2 enzyme. Cytosines were deaminated using the APOBEC enzyme, followed by PCR amplification and indexing. The libraries were checked on an Agilent bioanalyzer for their quality scores. All the libraries were pooled together in equimolar concentrations and sequenced on the NovaSeq 6,000 platform using S2 flow cell with 2 × 250 paired-end sequencing chemistry.

### 2.5 Sequencing data analysis

Demultiplexed samples were quality checked using the FastQC tool. Adapters and low-quality bases were trimmed using the Trim Galore tool and the sequencing quality was checked again after adaptor trimming. The trimmed reads were then aligned using the Bismark tool with the human reference genome (Hg38) sequence. Alignment was performed with default settings, and methylation extraction was performed using the Bismark's methylation extractor tool. The differential methylation analysis was conducted using the GeneDMRs package in R. CpG sites with coverage below 5 reads were excluded from further analysis. Differentially methylated genes and genomic features were identified using a logistic regression model with a minimum methylation difference threshold of 15% and an FDR-adjusted *p*-value < 0.05.

## 3 Results

### 3.1 Sample characteristics

After analyzing ROS levels in 50 samples each for normozoospermic and oligozoospermic groups, we selected a total of eight samples, four each from normozoospermic (presumptive fertile) and oligozoospermic infertile individuals for further analysis. Sample characteristics for oligozoospermic and normozoospermic infertile individuals are provided in Table 1. The mean age was comparable between cases and controls (Table 1), minimizing the effect of age on methylation difference. Sperm count, sperm concentration and sperm motility were significantly lower in the oligozoospermic group in comparison to the normozoospermic presumptive fertile group (Table 1).

**Table 1:** Clinical characteristics of patients.

Parameter	Oligozoospermic infertile (*N* = 4)	Normozoospermic presumptive fertile (*N* = 4)	Normal reference range (WHO 2021)
Age (years), mean ± SE	30.5 ± 0.87	31 ± 0.71	**-**
Sperm count (million/ejaculate)	20.3 ± 7.11	134.45 ± 37.4	≥39
Sperm concentration (million/mL)	8.75 ± 2.36	59 ± 13.2	≥16
Sperm motility (%)	51.5 ± 1.61	62.5 ± 7.5	≥42（PR + NP）

### 3.2 Semen samples have low and high ROS irrespective of sperm count

Intracellular ROS level in sperm was measured using the fluorescent dye H_2_DCF-DA through flow cytometry. On the basis of ROS values, both normozoospermic and oligozoospermic groups were divided into two sub-groups, i.e., high ROS and low ROS. In the normozoospermic group, the mean ROS values were 164 ± 34 in the low ROS (NLR) subgroup and 1,809 ± 754.11 in the high ROS (NHR) subgroup. Similarly, in the oligozoospermic group, the mean ROS levels were 230.5 ± 64.51 in the low ROS (OLR) subgroup and 1,968.5 ± 321.5 in the high ROS (OHR) subgroup (Figure 1). To date, no universally accepted threshold values have been established for categorizing intracellular ROS levels into low and high ROS groups. Therefore, group specific thresholds were selected to achieve a significant difference in ROS levels between the two groups. Accordingly, samples with mean ROS values ≤500 were classified as low ROS, whereas those with mean ROS values ≥1,000 were classified as high ROS. Samples with intermediate ROS levels (500–1,000) were excluded from further analysis to avoid potential overlap between the groups. Based on these criteria, eight samples representing distinct combinations of ROS levels and sperm counts were selected for genome-wide DNA methylation analysis.

**Figure 1:**
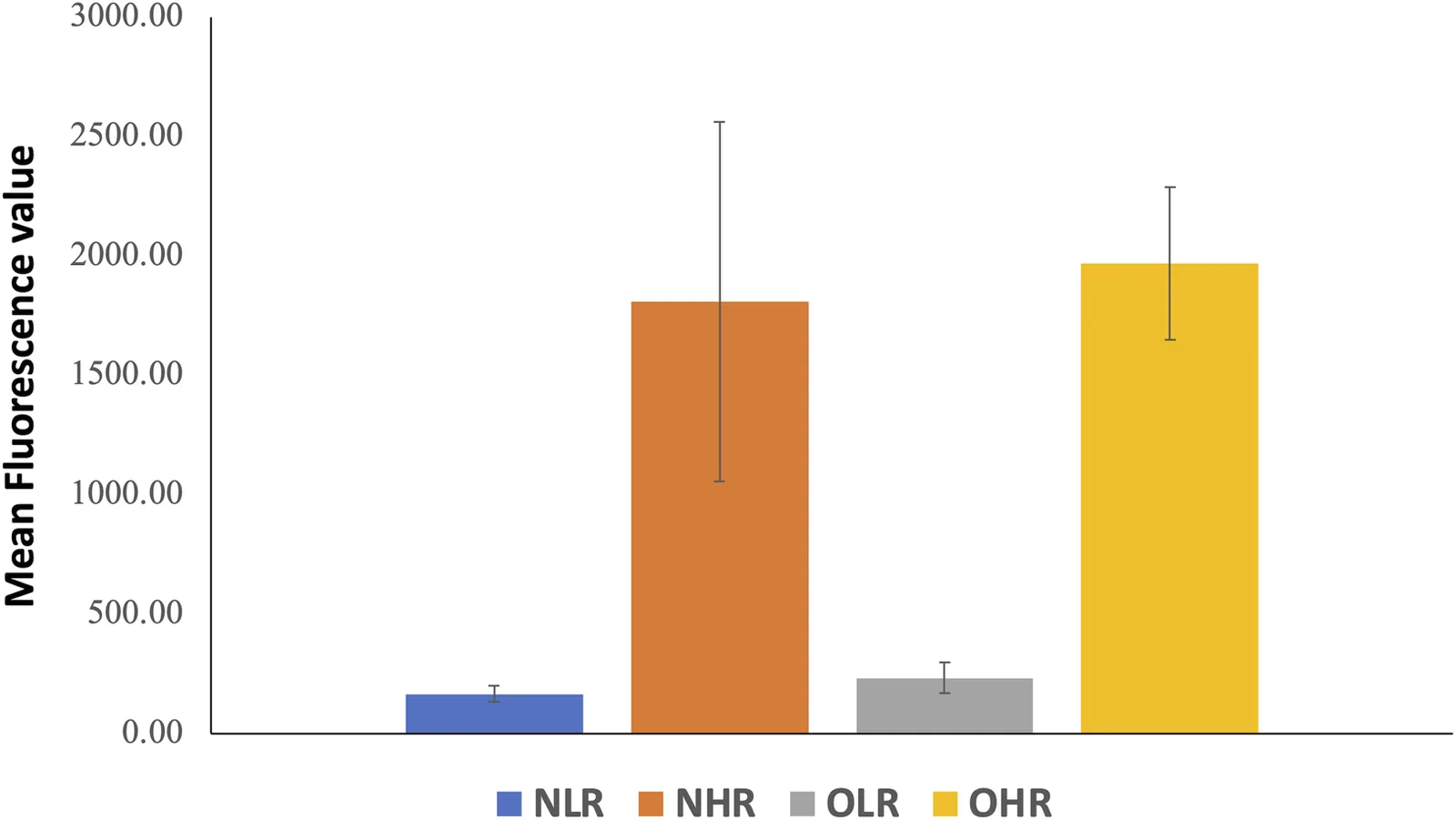
Bar graph showing the mean ROS values for the NLR, NHR, OHR and OLR subgroups.

### 3.3 Quality analysis of samples

The libraries for all samples were prepared using the same library preparation kit and sequenced in a single batch to minimize batch effects. Sequencing depth and mapping efficiency were assessed for all samples and are summarized in Supplementary Table S1. Sample quality analysis was performed to confirm the absence of somatic cell contamination. Somatic cell contamination was checked by assessing the methylation level of 14 CpG sites in the DLK1 gene, known to be differentially methylated between sperm and blood samples (12). All samples showed low methylation at these CpG sites, except for one sample showing hypermethylation at two out of 14 CpG sites (Supplementary Figure S1). This suggested high purity of sperm samples without significant somatic cell contamination. Bisulfite conversion efficiency for all samples was determined by analyzing the methylation in the CHH context across the whole genome, as suggested previously (13). The methylation levels in the CHH context ranged from 0.4% to 0.7% in all samples, indicating a high conversion efficiency of 99.3% to 99.6% (Supplementary Figure S2).

### 3.4 Chromosome-wise distribution of DMRs

DNA methylation alterations may not be spread equally across the whole genome. Therefore, we further analyzed if certain chromosomes showed a higher density of DMRs. For comparison, we normalised the number of DMRs by chromosome size (Mbp). Chromosome 21 was found to have the highest density of DMRs in all three comparison groups (Figure 2).

**Figure 2:**
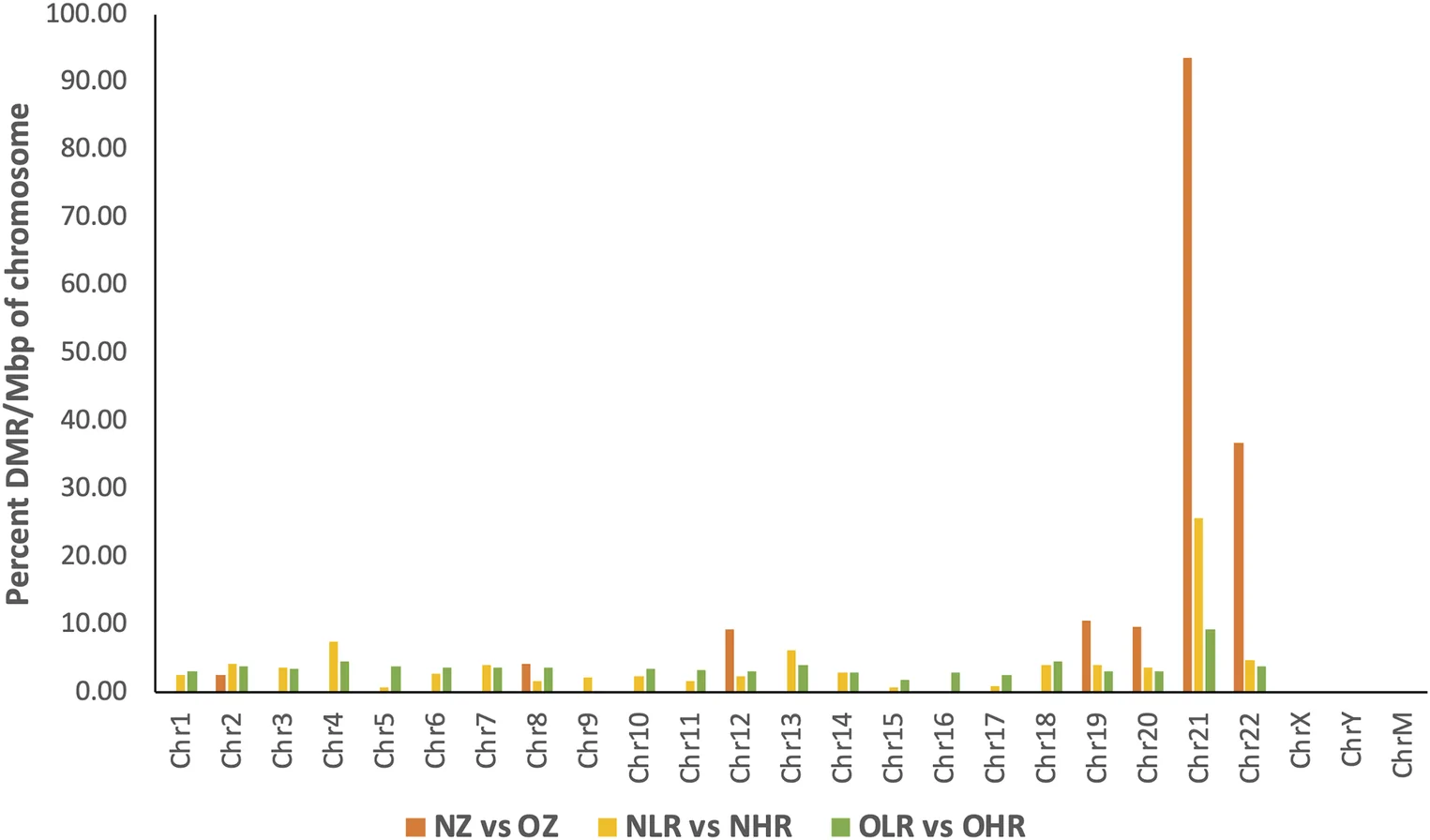
Chromosome-wise distribution of DMRs**.**

### 3.5 Global methylation shows minor differences

Global DNA methylation was assessed for individual samples in all groups. Mean DNA methylation across CpG sites was 74.75% in the normozoospermic low ROS group, 75.3% in the normozoospermic high ROS group, 75.9% in the oligozoospermic low ROS group, and 74.05% in the oligozoospermic high ROS group (Supplementary Figure S2). Mean global DNA methylation comparison across all CpGs showed no substantial difference between various comparison groups. However, CHG, CHH and CN/CHN sites showed different degrees of methylation across comparison groups, which did not directly correlate with ROS levels.

### 3.6 Oligozoospermic versus normozoospermic comparison showed low level of differential methylation

We began with a comparison of methylation patterns between oligozoospermic and normozoospermic individuals. Globally, the two groups did not differ in mean methylation significantly (Supplementary Figure S3). The correlation in methylation levels across samples was relatively moderate to high, with the correlation coefficient between samples ranging from 0.4 to 0.8 (Supplementary Figure S3). Using a volcano plot, we identified differentially methylated CpG (DMC) sites across varying methylation levels (5%, 10%, 15%, 20%, and ≥25%) with a significance threshold of *p* < 0.05 (Supplementary Figure S3). A broad view of the distribution of differentially methylated CpG sites across chromosomes showed prominent peaks on chromosomes 1 and 16, suggesting unequal distribution of DMCs across chromosomes (Supplementary Figure S3). Overall, this comparison identified differential methylation in 100 genes, with 47 genes being hypermethylated and 53 hypomethylated. The observed methylation differences ranged from 44.53% to −39.04% (Figure 3).

**Figure 3:**
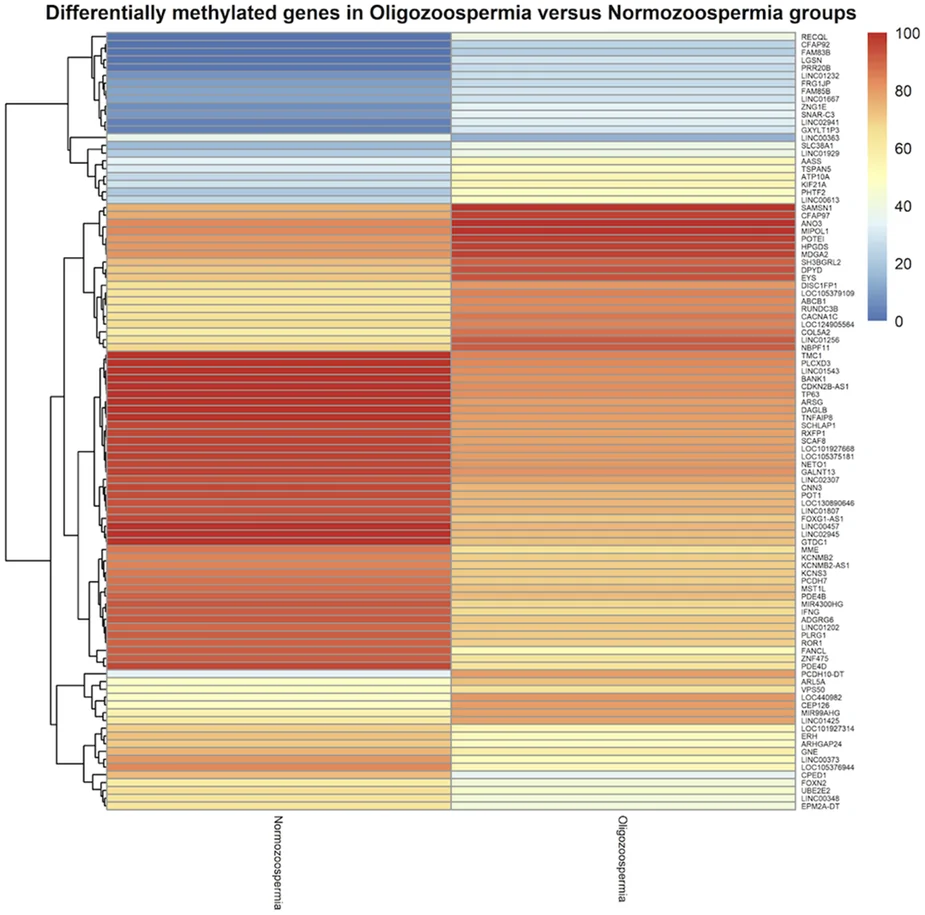
Heat map showing the differentially methylated genes between normozoospermic and oligozoospermic groups.

Subsequently, we analyzed various genomic features, including promoters, exons, introns, and transcription start sites (TSS), to identify the most affected regions in the abnormal spermatogenesis group. We observed 12 differentially methylated exons, including 5 hypermethylated (*GXYLT1P3, SNAR-C3, LINC01929, ZNF595* and *BAGE2*) and 1 hypomethylated (*IFNG*) exon-1 regions, and 5 hypermethylated and 1 hypomethylated other exons. Additionally, we identified 160 differentially methylated introns across 121 genes, with 23 hypermethylated and 28 hypomethylated intron-1 regions, and 47 hypermethylated and 62 hypomethylated other introns. We also observed 20 differentially methylated promoters, with 17 being hypermethylated (*ANKRD18B, BAGE2, CAST, CHEK2P2, FAM88B, FOXD4L4, GAGE2A, GPRIN2, GXYLT1P3, LOC102724874, PRR20B, SNAR-A10, SNAR-B1, LINC01667, SNAR-C3, CFAP92* and *FRG1FP*) and 3 being hypomethylated (*GLYATL1, ROR1-AS1* and *MST1L*). Further analysis of CpG islands revealed hypermethylation at 7 CpG islands, while 38 CpG shores were hypermethylated and 4 were hypomethylated (Figure 4).

**Figure 4:**
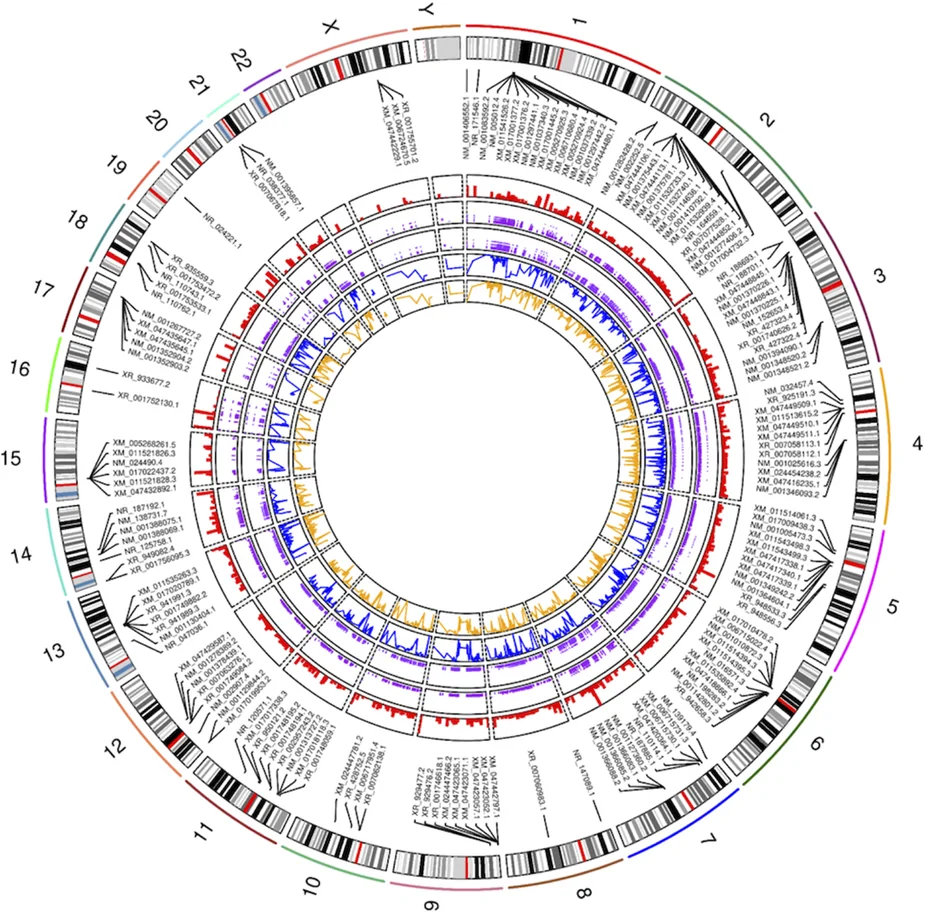
Comparison of OZ and NZ groups: circos diagram representing genome-wide distribution of DMCs. The circles, arranged from the outermost track to the innermost represent the chromosomes, differentially methylated genes, gene densities, CpG island densities, CpG island shore densities, and methylation levels.

### 3.7 Normozoospermic high ROS versus normozoospermic low ROS comparison showed moderate level of differences

Global methylation level was compared between the NLR and NHR groups, showing the NHR group to be hypermethylated in comparison to the NLR group (Supplementary Figure S4). The correlations in methylation levels across different samples were relatively high, with the correlation coefficient (r) between samples of 0.8 (Supplementary Figure S4). The volcano plot shows differentially methylated CpG sites across varying methylation levels of 5%, 10%, 15%, 20% and ≥25% with statistical significance (*p* < 0.05), showing more of hypomethylated CpG sites (Supplementary Figure S4). Similarly, genome-wide distribution analysis of differentially methylated CpG sites showed the highest number on chromosomes 1 and 16 (Supplementary Figure S4). This suggests that these chromosomes may contain key genomic regions experiencing substantial methylation alterations in relation with ROS levels. Overall, this comparison showed differential methylation across 538 genes (including 217 hypomethylated and 321 hypermethylated in normozoospermic high ROS group) with the level of differential methylation ranging from 56.06 to −47.06% (Figure 5). Further in-depth analysis showed 114 differentially methylated exons across 108 genes, including 25 hypermethylated and 19 hypomethylated exon-1 regions, and 36 hypermethylated and 34 hypomethylated other exons. We also identified 1,084 differentially methylated intronic regions among 809 genes, with 239 hypermethylated and 150 hypomethylated intron-1 regions, as well as 397 hypermethylated and 298 hypomethylated other introns. We also observed 53 differentially methylated promoters, with 31 being hypermethylated and 22 being hypomethylated. This suggests that ROS-induced methylation changes are not confined to gene promoter regions but extend across coding and non-coding regions, which could have broad implications for gene regulation and transcript stability. Further analysis of CpG islands revealed hypomethylation across 7 CpG islands, while 39 CpG shores were hypermethylated and 62 were hypomethylated (Figure 6).

**Figure 5:**
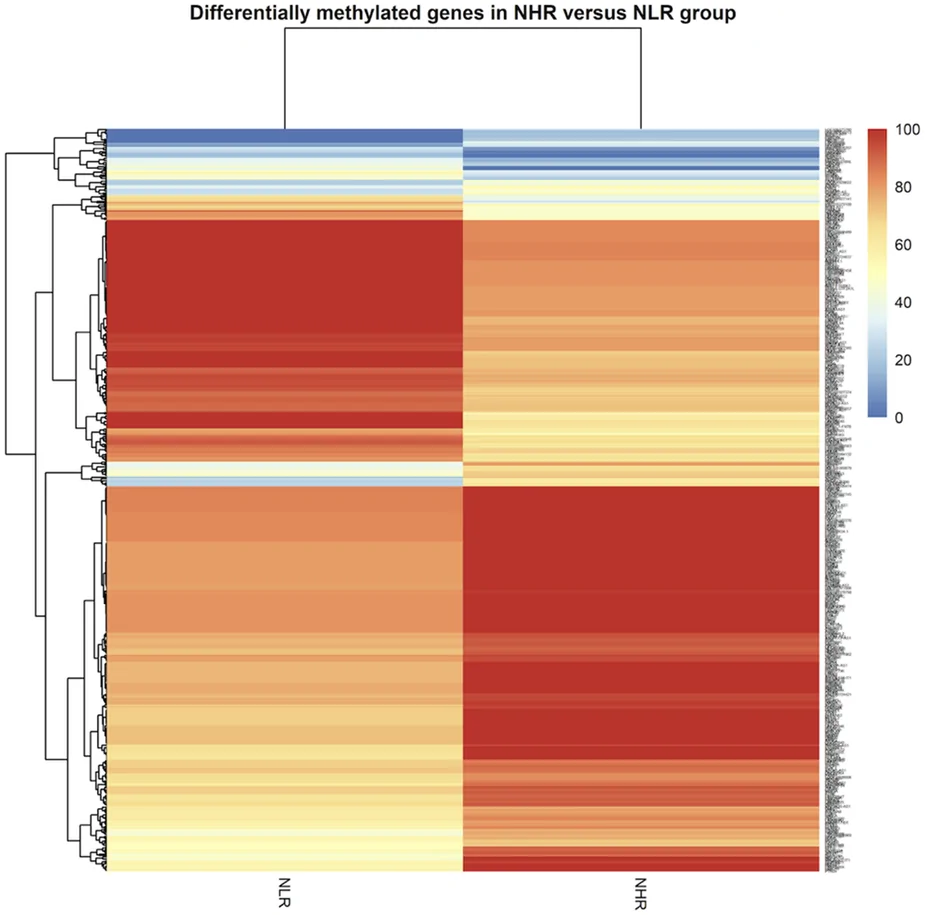
Heat map showing the differentially methylated genes between the NHR and NLR groups.

**Figure 6:**
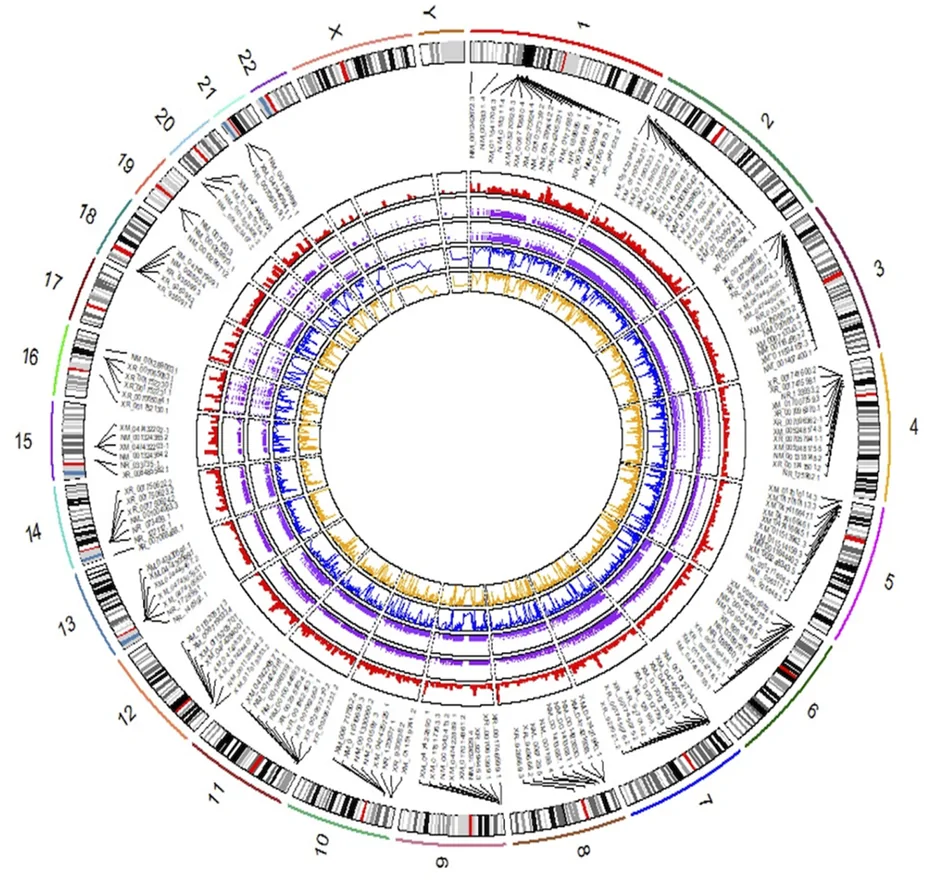
Comparison of NHR and NLR groups: circos diagram representing genome-wide distribution of DMCs. The circles, arranged from the outermost track to the innermost represent the chromosomes, differentially methylated genes, gene densities, CpG island densities, CpG island shore densities, and methylation levels.

### 3.8 Oligozoospermic high ROS versus oligozoospermic low ROS comparison showed high level of differential methylation

Global methylation level showed the OHR group to be hypomethylated in comparison to the OLR group (Supplementary Figure S5). The correlations in methylation levels across different samples were relatively high with the correlation coefficient between samples in the range of 0.4–0.8 (Supplementary Figure S5). The volcano plot showed differentially methylated CpG sites across varying methylation levels of 5%, 10%, 15%, 20% and ≥25% with statistical significance of 0.05 with major CpG site being hypermethylated (Supplementary Figure S5). The chromosome-wise distribution of differentially methylated CpG sites across chromosomes again showed the highest number of CpGs on Chromosomes 1 and 16 (Supplementary Figure S5). Altogether, 1,755 genes were differentially methylated, including 620 hypermethylated and 1,135 hypomethylated genes with the level of methylation ranging from 74.12 to −74.66% (Figure 7).

**Figure 7:**
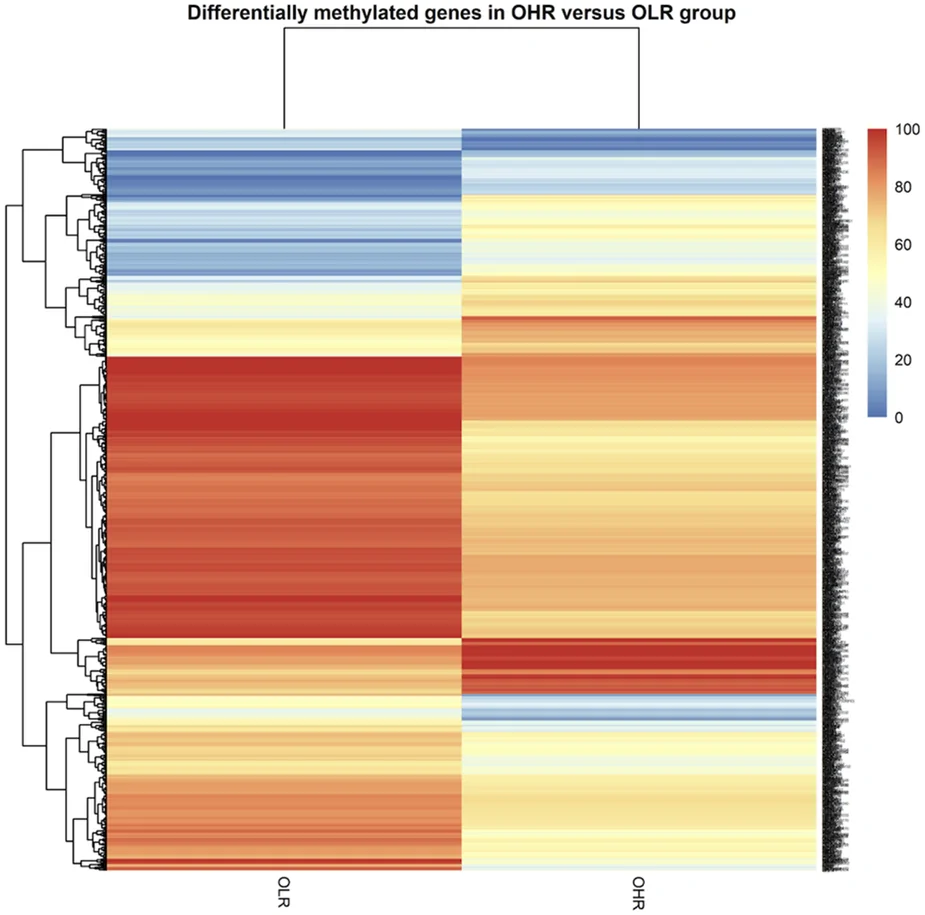
Heat map showing the differentially methylated genes between the OHR and OLR groups.

Further, while investigating the genomic features, we identified 5,083 differentially methylated exons across 4,256 genes, including 656 hypermethylated and 1,176 hypomethylated exon-1 regions, alongside 1,024 hypermethylated and 2,227 hypomethylated other exons. Introns were also significantly impacted, with a total of 10,581 differentially methylated introns across 6,933 genes, including 1,045 hypermethylated and 1,992 hypomethylated intron-1 regions, as well as 2,142 hypermethylated and 5,042 hypomethylated other introns. Additionally, we observed 2,701 differentially methylated promoters, with 1,234 being hypermethylated and 1,467 being hypomethylated. Transcription start sites (TSS) were also found to be differentially methylated across 9 genes including 5 hypermethylated (including *MIR100HG, MIR3667HG, RNA5S9, LINC00200* and *DTNA)* and 4 hypomethylated (including *MIR4797, ARHGAP26-AS1, BIN1 and OR7E91P*). CpG island-wise analysis showed 536 hypermethylated and 167 hypomethylated CpG islands, while 3,576 CpG shores were hypermethylated and 2,732 were hypomethylated (Figure 8). This suggest overall hypomethylation in the OHR group in comparison to the OLR group.

**Figure 8:**
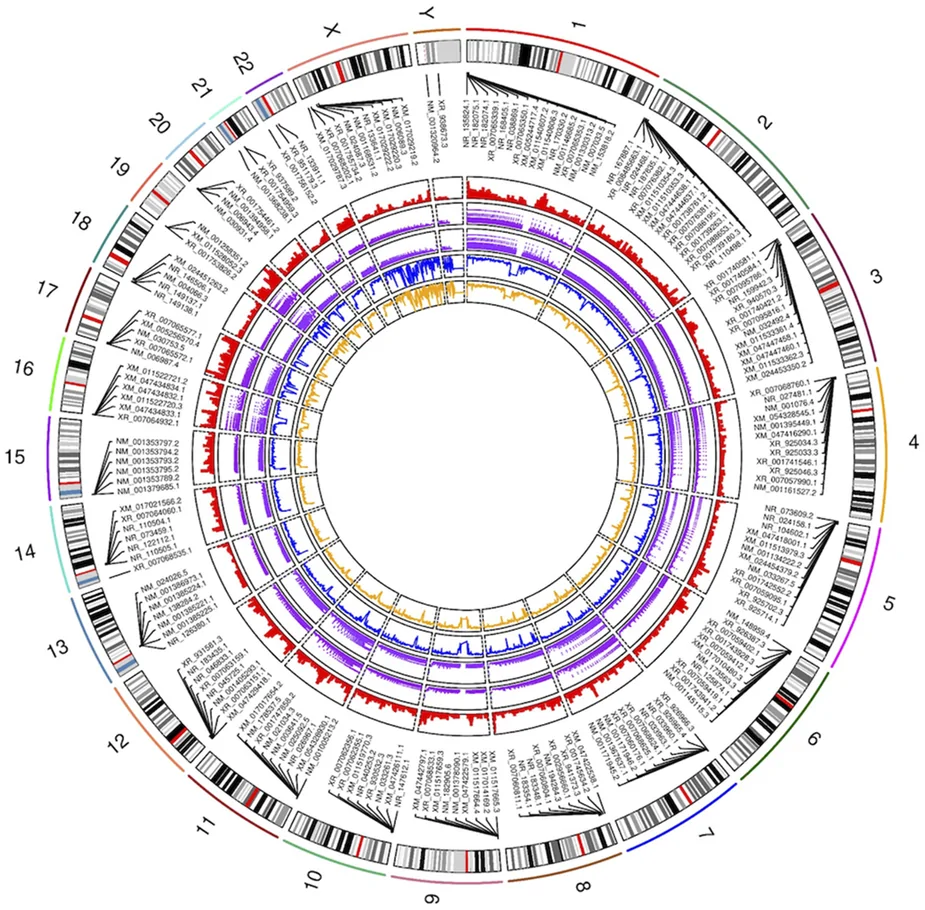
Comparison of OHR and OLR groups: circos diagram representing genome wide distribution of DMCs. The circles, arranged from the outermost track to the innermost circle, represent the genome chromosomes, differentially methylated genes, gene densities, CpG island densities, CpG island shore densities, and methylation levels.

### 3.9 Gene ontology for differentially methylated genes

The genes which were found to be differentially methylated as whole, Exon-1 and Intron-1 were further subjected to gene ontology enrichment first using the string database and if needed using the R-tool.

In the OZ versus NZ group comparison, 67 hypermethylated and 59 hypomethylated genes did not produce any output for gene ontology using string database, then we analyzed it using R-tool which showed that the hypermethylated genes are majorly involved in phospholipid translocation and transport, lipid translocation, regulation of membrane lipid distribution, amino acid biosynthetic process and non-proteinogenic amino acid biosynthetic process (Supplementary Figure S6) while hypomethylated genes showed enrichment for single biological process, i.e., gland morphogenesis (Supplementary Figure S6). Thus, the overall comparison between the two groups did not highlight genes involved in reproduction.

In the normozoospermic sub-group comparison (NHR versus NLR), 448 hypermethylated and 312 hypomethylated genes were mapped for gene ontology using the string database. Hypermethylated genes showed enrichment of genes in cellular process, biological regulation, regulation of cellular process, cell communication, signalling and many other biological processes (Supplementary Figure S7), while hypomethylated genes showed enrichment in localization, signalling, system process, muscle contraction, cardiac muscle contraction and many others (Supplementary Figure S7). In this comparison as well, no reproduction related genes were found to be differentially methylated.

In the oligozoospermic sub-group comparison (OHR versus OLR), 2,694 hypermethylated and 3,971 hypomethylated genes were mapped for gene ontology using the string database. Hypermethylated and hypomethylated genes showed enrichment in the regulation of biological, cellular, metabolic and cellular processes (Supplementary Figure S7). Interestingly, hypomethylated genes in this comparison were also found to be involved reproductive process, with more than 100 genes found enriched in reproduction, positive regulation of biological and cellular process, and reproductive processes (Figure 9). Further within the reproductive processes, it showed enrichment of more than 25 genes in male gamete generation and spermatogenesis, 22 genes in cell differentiation (Figure 9), and 36 genes in embryo development (Figure 9). This way genes involved in reproduction were found to be significantly hypomethylated, particularly in the oligozoospermic high ROS group.

**Figure 9:**
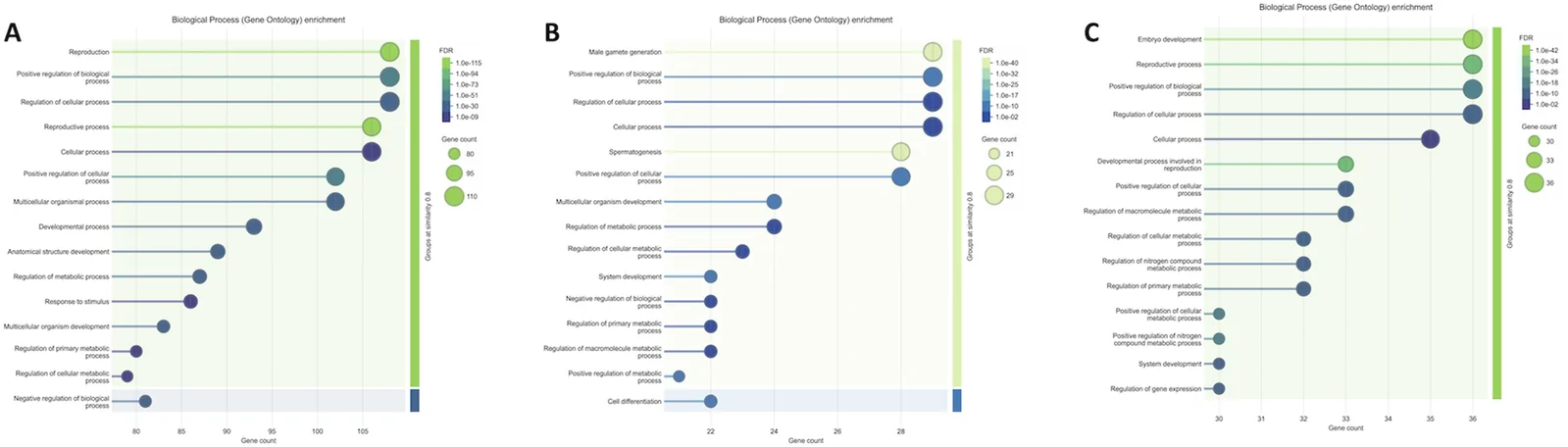
Gene ontology (GO) enrichment analysis highlighting reproductive processes in hypomethylated genes from the OHR vs OLR comparison. **(A)** Enrichment of hypomethylated genes in reproductive processes, **(B)** Subcategory analysis within reproductive processes showing enrichment of genes associated with male gamete generation, spermatogenesis, and cell differentiation, and **(C)** Enrichment of hypomethylated genes involved in embryo development processes.

## 4 Discussion

Assisted reproduction brought a paradigm shift in the treatment of infertility. Since the inception, over 10 million children have taken birth with the help of assisted reproduction. Men with even one sperm in their testis can hope to father a biological child. After the first generation of IVF born children reached the age of 40, it was realized that they have a higher incidence of developmental disorders (14–19). In the beginning, this notion was not well accepted; however, longitudinal studies later on provided concrete data that established the adverse impact of assisted reproduction on the long-term health of individuals born through assisted reproduction (20, 21). Case control studies have supported a higher prevalence of imprinting disorders in children born through ART (22). A report from Sweden reported a relatively higher incidence of imprinting disorders in children born through assisted reproduction, despite adjustment for confounding factors (23). After realizing the impact of assisted reproduction on a higher incidence of development disorders, the next pertinent question was to understand the mechanisms that operate in raising this incidence to a high level. The *in-vitro* culture environment, including culture-media composition and other ART-related conditions, has been shown to significantly affect DNA methylation (22). It was seldom emphasized that sperm produced by infertile men could be molecularly abnormal due to disturbed testicular molecular milieu. We analyzed sperm DNA methylome in oligozoospermic infertile individuals and identified a number of differentially methylated regions throughout the genome (8). This was the first study reporting the molecular abnormalities in oligozoospermic infertile sperm samples.

It has been emphasized by a number of studies on environmental epigenetics that aberrant DNA methylation can affect developmental origin of health and diseases (DOHaD) whereby an environmental factor brings significant epigenetic adaptations, altering the future health trajectory and disease state of an individual (24–26). In oligozoospermic individuals, exposure to certain genetic or environmental factors brings gene expression changes, which translate into poor sperm production and infertility. It is unarguably acceptable that poor sperm production or infertility would link to some critical factor missing or compromised in the testis. Since DNA methylation is a critical regulator of gene expression, altered DNA methylation in germ cells may account for poor sperm production. Irrespective of the genetic or environmental origin of this alteration, altered DNA methylation makes its way into sperm that are naturally infertile. Assisted reproduction provides an opportunity to these individuals to father children using otherwise ‘rejected’ sperm by natural mechanisms that keep a check on the quality of gametes. Therefore, assisted reproduction offers a chance to promote ‘DOHaD’ originating in the father, which is now transferred to the next generation. Looking at significant alterations in sperm DNA methylation in infertile men, we believe the developmental issues seen in children born through assisted reproduction could be the result of DNA methylation changes carried by their sperm, which might be further complicated by exposure to the *in vitro* environment.

In the present study, we made an effort to understand the cause of DNA methylation changes in the sperm of oligozoospermic and normozoospermic individuals. In the case of normozoospermic individuals, ROS level did not alter DNA methylation to a significant level, which could perhaps be due to a number of other safeguarding mechanisms still effective in these individuals. On the other hand, in oligozoospermic individuals with high ROS levels, we observed a deep impact on DNA methylation, resulting in altered methylation spread across 1,755 genes and 18,374 gene features, including 5,083 exons, 10,581 introns, 2,701 promoter and 9 transcription start sites regions. Previous studies have established an association between oxidative stress and altered methylation of the imprinted genes IGF2 and H19 (27). Darbandi et al. reported that increased ROS levels were positively correlated with IGF2 methylation and negatively correlated with H19 methylation, indicating that oxidative stress may influence the epigenetic regulation of these imprinted loci in human sperm (27). ROS may react with the DNA methyl group converting it to their oxidized form, which may ultimately get de-methylated. Interestingly, we observed a majority of differentially methylated regions in the oligozoospermic group to be hypomethylated, suggesting the impact of high ROS levels on them. The pronounced impact of elevated ROS level in oligozoospermic individuals may be attributed to defective chromatin packaging often associated with oligozoospermic individuals (27–29). It appears that high ROS in oligozoospermic individuals would react with DNA methyl groups, leading to DNA hypomethylation. Interestingly, the GO analysis showed impact on reproductive processes only in the case of hypomethylated regions in the oligozoospermic group (high ROS versus low ROS), suggesting that oxidative stress mediated DNA hypomethylation might affect spermatogenesis in oligozoospermic individuals with high ROS.

There is significant supporting evidence that oxidative stress results in altered DNA methylation. The presence of 8-OHdG at CpG sites (30–32) and O6-methylguanine (33) inhibits the methylation of adjacent cytosine residues, resulting in DNA hypomethylation. Aberrant DNA methylation (hypomethylation in particular) has been reported to be more common in infertile men in comparison to normozoospermic fertile men (34–38). More interestingly, a reduction in sperm DNA methylation (hypomethylation) has also been linked with a reduction in IVF pregnancy rates (36, 39, 40). Further, the relationship between oxidative stress and DNA methylation in somatic cells is already well established as the presence of ROS induced DNA adducts has been linked with compromised DNA methyltransferase activity (31, 32). Some other previous studies reported a negative correlation between DNA fragmentation and DNA methylation (39, 41); however, none of these measured ROS levels in their infertile samples. A previous study also reported a significant inverse relationship between ROS and sperm DNA methylation in infertile men (42).

The strongest evidence that DNA methylation abnormalities can directly cause human disease comes from imprinting disorders such as Beckwith–Wiedemann syndrome (BWS) and Silver–Russell syndrome (SRS) (43, 44). In BWS, gain of methylation at the H19/IGF2 imprinting control region (IC1) or loss of methylation at the KCNQ1OT1:TSS differentially methylated region (IC2) on chromosome 11p15.5 leads to dysregulated expression of IGF2, H19, and CDKN1C, resulting in somatic overgrowth and tumor predisposition (44, 45). Conversely, in SRS, loss of methylation at IC1 reduces IGF2 expression and causes severe fetal and postnatal growth restriction (46, 47). Importantly, many affected individuals harbor only epimutations, without any underlying DNA sequence alterations, establishing aberrant DNA methylation as a direct cause of human disease (43, 48). We found several imprinted genes to be differentially methylated in oligozoospermic high ROS and low ROS comparison. In contrast, no differentially methylated imprinted genes were found in normozoospermic high ROS and low ROS comparison or in the overall oligozoospermic vs normozoospermic comparison. Among the differentially methylated imprinted genes identified in the OHR versus OLR comparison, hypermethylation within promoter regions was observed in MEST, PLAGL1, and GNAS-AS1. IGF2-AS exhibited hypermethylation in exon 1. MEST, PEG10, and MEG3 showed hypermethylation in intron 1, whereas MAGI2-AS3 was hypomethylated. Hypermethylation in other exonic regions was identified in IGF2-AS, PEG10, and GNAS-AS1. Within other intronic regions, PEG3, MEG3, and GNAS-AS1 exhibited hypermethylation, while KCNQ1, PLAGL1, and MAGI2-AS3 exhibited hypomethylation. Apart from these known regions of altered methylation, there could be other genomic regions contributing to these or other developmental disorders.

Interestingly, Tunc and Tremellen (2009) also reported a significant improvement in DNA methylation upon antioxidant therapy in patients for three months, further suggesting that ROS was the cause behind DNA hypomethylation (42). Therefore, a careful assessment before undertaking assisted reproduction in oligozoospermic individuals might be worth considering. It can be argued that DNA methylation analysis should precede such decisions; however, if further studies with larger sample sizes replicate our findings, a simple translation of high ROS into high epigenetic alterations in oligozoospermia may be sufficient to decide the course of infertility treatment. However, further research is required on the extent of developmental abnormalities that can be attributed to sperm DNA methylation. Apart from a higher incidence of developmental disorders, it would also be worth investigating if ART born children face relatively higher health issues in long-term. Further studies on bigger sample sizes are required to validate our findings.

In conclusion, the present study analyzed the DNA methylation differences between low and high ROS oligozoospermic and normozoospermic groups. We conclude that in normozoospermic individuals, ROS do not penetrate to affect DNA methylation significantly. On the other hand, in the case of oligozoospermic individuals, high ROS levels cause significant changes in DNA methylation, resulting in hypomethylation of thousands of DNA regions. Interestingly, these hypomethylated regions span reproductively important genes. Since oligozoospermic individuals often undergo assisted reproduction, such massive DNA methylation changes might contribute to the higher incidence of development disorders in children born with the help of assisted reproduction. Nevertheless, further proof is required to prove the real impact of sperm DNA methylation on embryonic development and on health trajectory of upcoming generations as most of the epigenetic changes get erased at the time of embryonic development (13, 49). Due to its significant impact on DNA methylation, ROS analysis before assisted reproduction would be of high value. A major limitation of our study is the relatively small sample size in each group. If further studies replicate our findings, ROS analysis might find value in deciding upon the quality of donor sperm for assisted reproduction. Overall, our findings suggest not to disregard ROS values during assisted reproduction just because ICSI can help bypass the natural barrier to reproduction in oligozoospermic individuals. Understanding molecular changes in infertile sperm would help us improve the value of assisted reproduction by ensuing quality apart from ensuring parenthood.

## Data Availability

The data generated in this study have been deposited in the NCBI repository and can be accessed with BioProject no. PRJNA1514642. The data can be accessed here: https://www.ncbi.nlm.nih.gov/sra/PRJNA1514642.
