# Supplementary material for: Oligozoospermic infertile sperm with high ROS are more prone to epigenetic alterations
Source: Front Reprod Health. 2026 Sep 23;8:1933266. doi: 10.3389/frph.2026.1933266 (PMC13642678; doi:10.3389/frph.2026.1933266)
Supplement: Supplementary file 1 [file Table1.docx]

**Table S1.** Sequencing quality metrics for samples included in the study.

| **Sample ID** | **Group** | **Total Reads (Million)** | **Q30 (%)** | **Mapping Efficiency (%)** |
| --- | --- | --- | --- | --- |
| NLR-R | NLR | 115.74 | 89.3 | 65.7 |
| NLR-N | NLR | 243.65 | 89.6 | 66.9 |
| NHR-D | NHR | 161.07 | 89.5 | 65.5 |
| NHR-Sa | NHR | 223.18 | 89.7 | 63 |
| OLR1 | OLR | 893.83 | 90.5 | 58.4 |
| OLR-100S | OLR | 218.96 | 88.8 | 66.3 |
| OHR1 | OHR | 328.38 | 90.5 | 52.5 |
| OHR-Am | OHR | 261.85 | 89.8 | 67.3 |
